# Overexpression of phosphatidylinositol 4-kinase type IIIα is associated with undifferentiated status and poor prognosis of human hepatocellular carcinoma

**DOI:** 10.1186/1471-2407-14-7

**Published:** 2014-01-06

**Authors:** Adeodat Ilboudo, Jean-Charles Nault, Hélène Dubois-Pot-Schneider, Anne Corlu, Jessica Zucman-Rossi, Michel Samson, Jacques Le Seyec

**Affiliations:** 1 INSERM, UMR-1085, Institut de Recherche Santé Environnement & Travail (IRSET), F-35043, Rennes, France; 2 INSERM, UMR-991, Liver Metabolisms and Cancer, F-35033, Rennes, France; 3 Université de Rennes 1, F-35043, Rennes, France; 4 Fédération de Recherche BioSit de Rennes, F-35043, Rennes, France; 5 INSERM, UMR-674, Génomique fonctionnelle des tumeurs solides, IUH, Paris F-75010, France; 6 Université Paris Descartes, Labex Immuno-oncology, Sorbonne Paris Cité, Faculté de Médecine, Paris, France

**Keywords:** Hepatocellular carcinoma, PI4KA, Biomarker, Prognosis

## Abstract

**Background:**

Hepatocellular carcinoma (HCC) is a particularly severe disease characterized by a high rate of recurrence and death even after surgical resection. Molecular characterization of HCC helps refine prognosis and may facilitate the development of improved therapy. Phosphatidylinositol 4-kinases have recently been identified as cellular factors associated with cancer. Also, phosphatidylinositol 4-kinase type IIIα (PI4KA) is necessary for the propagation of the hepatitis C virus, a major etiological factor for HCC.

**Methods:**

Reverse transcription, quantitative real-time PCR was used to assay PI4KA mRNA. The expression levels were investigated both in a collection of molecularly and clinically characterized hepatic tissues from 344 patients with diverse liver diseases and in human hepatocyte cell lines whose proliferative and differentiation status was controlled by specific culture conditions. Analytical microarray data for 60 HCC and six normal liver tissue samples were exploited to study correlations between PI4KA mRNA levels and cell proliferation markers *in vivo*. Postoperative disease-specific survival and time to recurrence in a set of 214 patients with HCC were studied by univariate and multivariate analyses.

**Results:**

PI4KA mRNA was more abundant in HCC than normal healthy tissues. This upregulation correlated significantly with both poor differentiation and the active proliferation rate in HCC. These associations were confirmed with *in vitro* models. Moreover, patients with HCC who had been treated by surgical resection and had higher PI4KA mRNA concentrations in their tumor tissue exhibited a higher risk of tumor recurrence (median time: 20 months versus 49 months, P = 0.0012) and shorter disease-specific survival (first quartile time: 16 months versus 48 months, P = 0.0004). Finally, the abundance of PI4KA mRNA proved to be an independent prognostic marker of survival for cases of HCC (hazard ratio = 2.36, P = 0.0064).

**Conclusions:**

PI4KA mRNA could be used as a new molecular marker to improve established prognostic models for HCC. These findings also indicate possible new lines of research for the development of innovative therapeutic approaches targeting PI4KA.

## Background

Liver cancers are the third leading cause of death by cancer worldwide, and are the sixth most common group of malignancies [1]. Hepatocellular carcinoma (HCC) is the most common primary cancer of the liver (70-80%), more frequent than cholangiocarcinoma, and is more frequent in men than in women [2]. It rarely occurs in normal liver. In western countries, HCC mostly affects patients already suffering from cirrhosis due to chronic alcohol intake, or to chronic hepatitis B virus (HBV) or hepatitis C virus (HCV) infections [3]. Some cases of HCC emerge by malignant transformation of hepatocellular adenomas (HCA). HCA are benign hepatocellular tumors that develop mostly in otherwise normal liver in women taking oral contraception [4]. Classifications for HCA and HCC, based on their molecular signatures, have been established to refine prognosis and to facilitate work to develop targeted therapies. Thus, a genotype/phenotype classification identified five different molecular subgroups of HCA: (i) hepatocyte nuclear factor 1 homeobox A (HNF1A) mutated, (ii) inflammatory, (iii) catenin (cadherin-associated protein) beta 1 (CTNNB1) mutated, (iv) inflammatory and CTNNB1 mutated, and (v) unclassified. The presence of mutations in the CTNNB1 gene is a factor for poor prognosis, as they are associated with a high risk of malignant transformation of HCA to HCC [5,6]. Several transcriptomic classifications of HCC provide evidence of the substantial genetic and phenotypic heterogeneity of this tumor type [7]. One of these classifications individualizes six molecular subgroups (G1 to G6) related to clinical and pathological features [8]. However, the mechanisms underlying the molecular and phenotypic differences between HCCs remain to be deciphered.

There has been growing interest in phosphatidylinositol 4-kinase type IIIα (PI4KA) and its involvement in liver disease. We and others have shown that this enzyme is required for the propagation of HCV, one of the main etiological factors of HCC [9-13]. Four different phosphatidylinositol 4-kinases (PI4Ks) are expressed in human cells [14]. These isoenzymes (PI4KA, PI4KB, PI4K2A and PI4K2B) catalyze the phosphorylation of phosphatidylinositol (PtdIns) in the cytoplasmic face of cellular membranes, leading to the production of phosphatidylinositol 4-phosphate (PtdIns4P). Each isozyme displays a specific subcellular distribution. Thus, PI4KA is mainly found in the endoplasmic reticulum (ER). Its activity seems to regulate both the formation of ER exit sites [15,16] and the concentration of PtdIns4P in the plasma membrane [17]. PtdIns4P is a precursor of other phosphoinositides (PIs), generated by additional phosphorylation(s), involved in a wide range of cellular functions [18]. For example, cell migration and proliferation are controlled by PI-dependent signaling pathways involving phospholipase C (PLC) isozymes or phosphoinositide 3-kinases. Therefore, it is unsurprising that some cancers are associated with various types of deregulation in these signaling pathways, including those affecting PI4Ks [19].

The analysis of PI4KA expression in various liver diseases may therefore be informative. It may improve the molecular characterization of HCC, providing diagnostic and prognostic tools, and may even be useful to adapt and improve therapy. Moreover, the importance of PI4KA to the HCV life cycle makes it a potential therapeutic target. However, any treatment targeting PI4KA may be affected by its expression. In this context, we investigated PI4KA expression in large cohort of liver diseases. Because antibodies suitable for the detection of the endogenous protein by immunohistochemistry are not available, we used quantitative RT-PCR to assay PI4KA mRNA. We found that the PI4KA gene was more strongly expressed in HCC than in normal liver. This expression was correlated with the differentiation status. PI4KA also appeared to be an independent marker of an unfavorable prognosis in HCC.

## Methods

### Biological materials

All patients gave written consent as required by French law. This study was approved by our local IRB committees (CCPRB for “Comité Consultatif de Protection des Personnes dans la Recherche Biomédicale” Paris Saint Louis and CPP for “Comité de Protection des Personnes” Ouest V). Liver tissues were collected in French hospitals and immediately frozen in liquid nitrogen after surgical resection. The first library included a total of 344 liver samples with five normal and 339 pathological tissues (21 cirrhosis, 101 HCA and 217 HCC). Two additional normal tissue samples were used as calibrators for the relative levels of transcripts in samples as determined by quantitative RT-PCR. The molecular subtype of each of the 101 HCA (see Additional file 1 for clinical and molecular features) was determined according to an established molecular classification using gene mutation and immunohistochemistry staining [4-6]. The set of 217 HCC included in this study has already been extensively described (the main clinical, pathological and molecular features are presented in Additional file 2) [20]. All HCC were screened for TP53 and CTNNB1 mutations and were classified using the G1-G6 molecular classification as previously described [8,21]. The second cohort consisted of liver fragments from 31 patients who underwent surgical resection for hepatic metastases; these fragments were taken in macroscopically normal liver at a distance from the metastasis.

Huh-7.5.1 and HepaRG cell cultures were maintained as previously described and were subjected to specific differentiation protocols [22,23]. Huh-7.5.1 cells were seeded at a density of 6 × 10^4^ per cm^2^ in standard medium, consisting of complete DMEM (Life Technologies) supplemented with 100 U/ml penicillin (Life Technologies), 100 μg/ml streptomycin (Life Technologies), 2 mM L-glutamine (Life Technologies), 10 mM HEPES (Life Technologies), nonessential amino acids (Sigma-Aldrich) and 10% heat-inactivated fetal bovine serum (Hyclone, Logan, UT, USA). When the culture reached 95% confluence, defined as day 0 (D0), the standard culture medium was supplemented with 1% dimethyl sulfoxide (DMSO, Sigma-Aldrich) for 6 consecutive days. Cells were collected on days 0, 1, 3 and 6 (D0, D1, D3 and D6, respectively). HepaRG cells were seeded at a density of 2.7 x 10^4^ per cm^2^ on day 0 and maintained for two weeks in William’s E medium (Life Technologies) supplemented with 100 U/ml penicillin (Life Technologies), 100 μg/ml streptomycin (Life Technologies), 5 μg/ml insulin (Sigma-Aldrich), 50 μM hydrocortisone hemisuccinate (Roussel) and 10% fetal bovine serum (Hyclone, Logan, UT, USA). Then, the culture medium was or was not supplemented with 2% DMSO (Sigma-Aldrich) for two additional weeks. Cells were collected at days 4, 15 and 30 post-seeding (D4, D15 and D30, respectively). D30- and D30+ indicate that cells were cultured without DMSO for 30 days or without DMSO for 15 days and then with 2% DMSO for 15 days, respectively.

### Quantitative RT-PCR and microarray analysis

DNA and RNA were purified with commercial kits (Qiagen). Quantitative RT-PCR and its data analysis were performed as previously described [24]. TaqMan gene expression assays (hs99999901_s1 and hs01021084m1, Applied Biosystems) were used to analyze 18S and PI4KA expression, respectively. The last assay detected both PI4KA mRNA variants referenced in GenBank (NM_058004.3 = Variant 1, NM_002650.2 = Variant 2) with no amplification of pseudogene products. The probe hybridizes at the exon-exon junctions 39–40 and 7–8 of variants 1 and 2, respectively. For absolute quantification, a plasmid (pCMV-SPORT6-hPI4KA, Open Biosystems) containing the PI4KA cDNA (BC018120) was used for calibration. The sequences of the other primers used are given in Additional file 3. The microarray data (60 HCC and 6 normal livers) have been extensively described previously [8] and are available on a public database (E-TABM-36).

### Statistics

Continuous data were compared using the non-parametric Mann–Whitney Test (two groups) or Kruskal-Wallis Test (more than two groups). Spearman or Pearson tests were used for correlation analysis according to sample size. We used the Mantel Cox log rank test and Kaplan Meier method to assess post-resection survival. Disease-specific survival is defined by the tumor-related death and patients who died of another etiology were censored. Recurrence-free survival was defined as the length of time after hepatectomy for HCC during which a patient survives with no sign of HCC. The last recorded follow-up visit was in February 2011. Univariate analysis using Cox models was performed to identify variables associated with disease-specific survival. Variables with a P value < 0.05 in the univariate analysis were entered into a Cox multivariate model. P values < 0.05 were considered as significant. Statistical analysis was performed using Graphpad Prism and R statistical software (http://www.R-project.org/).

This study adheres to the REMARK guidelines [25].

## Results and discussion

### PI4KA transcript levels in pathological human livers

**Figure 1:**
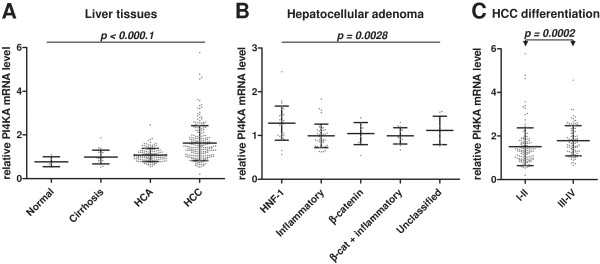
**PI4KA transcript abundance in human liver samples of various subtypes.** Scatter plots show the PI4KA mRNA levels in human liver tissues as assayed by RT-real time PCR. Values represent the gene expression of each sample relative to the mean value for two control samples from normal hepatic tissues. The 18S RNA levels were used for normalization. Means with standard deviations are indicated for each sample category. *P* values from Kruskal-Wallis tests (more than two groups, panels **A** and **B**; black line) or Mann–Whitney U-tests (two groups, panel **C**; black line with arrows) are indicated. **(A)** Comparison between normal hepatic tissue (n = 5), cirrhotic tissue (n = 21), and benign (HCA, n = 101) and malignant (HCC, n = 217) hepatocellular tumors. **(B)** Expression in different hepatocellular adenoma (HCA) groups subdivided into adenomas inactivated for HNF1A (n = 27), inflammatory adenomas (n = 44), β-catenin-activated adenomas (n = 10), inflammatory and β-catenin-activated adenomas (n = 13) and unclassified adenomas (n = 7). **(C)** Expression compared according to the differentiation grade of HCC grouped according to the Edmonson classification (Grades I-II, n = 118; Grades III-IV, n = 88).

A liver tissue library of 344 characterized samples was exploited to compare PI4KA transcript levels in normal and various pathological hepatic tissues (Figure 1A). There were no significant differences between normal and cirrhosis samples. PI4KA mRNA was slightly more abundant in HCA than in normal samples (1.4-fold; Mann–Whitney test: *P* = 0.0235). We therefore tested for differences between the different HCA subgroups classified according to their specific pathomolecular signature (Figure 1B) [4-6]. PI4KA mRNA levels were higher in HCA with mutations in HNF1A gene than other HCA subgroups. The HNF1A gene encodes the hepatocyte nuclear factor 1-alpha, involved in hepatocyte differentiation [26].

The level of PI4KA transcripts was 2.1 times higher (Mann–Whitney test: *P* = 0.0023) in HCC than normal reference samples (Figure 1A). The PI4KA mRNA values, however, did not differ significantly between HCC from patients with and without chronic HCV infection (data not shown). This is consistent with the observation that HCV promotes its replication by stimulating the activity of PI4KA but not the expression of its gene [27].

### Influence of differentiation/proliferative status of cancerous liver cells on PI4KA transcript levels

The overexpression of PI4KA in HCC could not be explained by HNF1A mutations: the frequency of these mutations was low in our HCC series (<4%) [28]. By contrast, the mutation of this hepatic differentiation factor was associated with upregulation of PI4KA mRNA in HCA. We therefore looked for an association between the differentiation state of HCC and amounts of the PI4KA transcript. We found that the less differentiated HCC, according to the Edmonson classification, showed stronger PI4KA expression (compare grades I-II with grades III-IV in Figure 1C).

**Figure 2:**
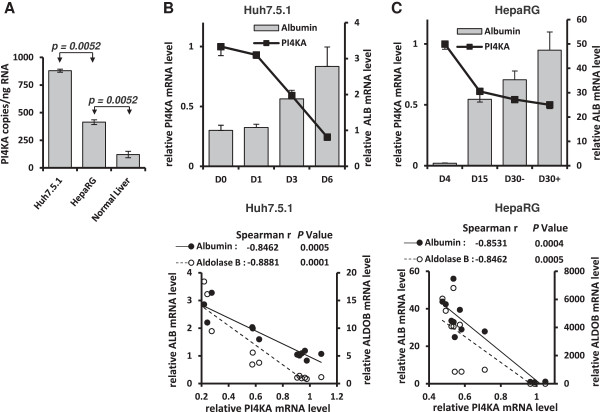
**PI4KA mRNA in *in vitro *models according to their hepatic differentiation state.** Determination by RT-real time PCR of copy numbers of PI4KA transcripts in sub-confluent cultures of Huh-7.5.1 (n = 3) or HepaRG (n = 3) and in normal human liver tissues (n = 31) **(A)**. *P* values from Mann–Whitney U-tests are indicated. Huh-7.5.1 **(B)** and HepaRG **(C)** cell lines were subjected to specific differentiation protocols over several days (see details in Methods section). PI4KA and hepato-specific (albumin and aldolase b) transcripts were assayed by RT-real time PCR at the time points indicated, in three independent experiments. Results are expressed in amounts relative to those at the first time point. The mRNA levels of the succinate dehydrogenase complex, subunit A (SDHA) were used for normalization. The top panels show the comparative expression of PI4KA and albumin. The bottom panels present the correlations between PI4KA and albumin or aldolase B expression levels. Spearman’s rank order coefficients and *P* values are indicated above the graphs.

To study the link between hepatic differentiation and PI4KA mRNA level further, we used two well-established *in vitro* models based on HCC derived cell lines (Huh-7.5.1 derived from Huh-7 [23]; and HepaRG) whose differentiation state can be controlled [22,29]. We compared the absolute amounts of PI4KA mRNA in Huh-7.5.1 cells, HepaRG cells and normal liver tissue. Normal human hepatic specimens, from a cohort different from that used in the analysis described above (presented in Figure 1), contained an average of 121.71 ± 28.59 PI4KA cDNA copies/ng of total RNA (Mean ± SD); HepaRG and Huh-7.5.1 cells, both at proliferative stages, had respectively 3.4-fold and 7.2-fold more PI4KA transcripts (Figure 2A). Thus, as in HCC from which these cell lines derive, a similar up-regulation of PI4KA transcripts was detected. Furthermore, the quantity of this mRNA was highest in the most dedifferentiated cell line (Huh-7.5.1). By applying their specific differentiation procedures, both cell lines gradually acquired a more differentiated hepatocyte state, as evidenced by the up-regulation of the liver-specific ALDOB gene encoding aldolase B and ALB gene encoding albumin. By contrast, the PI4KA transcripts levels gradually decreased during differentiation (Figure 2B and C, top panels). Spearman rank analysis showed that PI4KA mRNA quantities in Huh-7.5.1 and HepaRG cells were negatively correlated with the ALDOB and ALB mRNA quantities (Figure 2B and C, bottom panels). Given that the differentiated state of both cell lines is clearly associated with a decline in proliferative activity [29,30], our data indicate that an increase in the number of PI4KA transcripts correlates with hepatic dedifferentiation and active proliferation.

**Figure 3:**
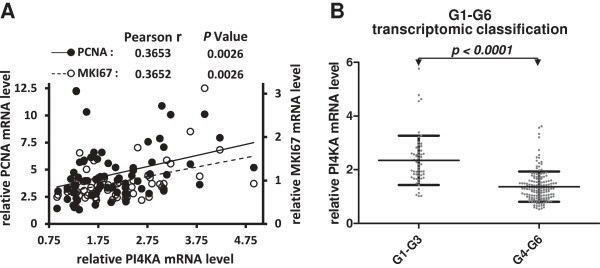
**Correlation between PI4KA expression level, proliferative markers and molecular status of HCC.** A correlation study between PI4KA expression levels and PCNA or MKI67 expression levels was performed in 60 HCC and 6 normal liver tissues analyzed with the HG-U133A Affymetrix GeneChip™ microarray. Pearson’s rank order coefficients and *P* values are indicated for the correlations **(A)**. Scatter plots show the PI4KA mRNA as assayed by RT-real time PCR in HCC specimens stratified according to transcriptomic classification (G1-G3, n = 58; G4-G6, n = 149) **(B)**. Values represent the gene expression of each sample relative to the mean value for two control samples from normal hepatic tissues. The 18S RNA levels were used for normalization. Means with standard deviations are indicated for each sample category. The *P* value from a Mann–Whitney U-test is indicated.

High mitotic activity and proliferation rate are also frequently associated with poor differentiation in cancer. We therefore tested for a relationship between PI4KA expression and two commonly used markers of cell proliferation (proliferating cell nuclear antigen [PCNA] and MKI67) in a series of 60 HCC and six normal liver tissue samples analyzed with the HG-U133A Affymetrix GeneChip™ microarray. PI4KA expression was partly but significantly correlated with PCNA and MKI67 expression in HCC (Figure 3A and B). This was corroborated by the observation that the level of PI4KA mRNA was higher in HCC classified in G1-G3 subgroups than in HCC classified in G4-G6 subgroups (Figure 3B). Indeed, the G1-G3 subgroups are known as the proliferative subclasses because they display transcriptomic dysregulation of cell cycle genes [8,31]; they are also typically enriched in poorly differentiated HCC (66% of HCC in G1-G3 subgroups have an Edmonson grade III-IV versus 35% of HCC in G4-G6 subgroups, *P* = 0.0001, Fisher’s exact test), such as those carrying TP53 mutations and those with high level of serum AFP, both factors correlating with stronger expression of PI4KA (Additional file 4). Thus, *in vivo* and *in vitro* data sets were consistent and suggest that the quantity of PI4KA transcripts is related to the differentiation status and the proliferation rate of tumors.

While PI4K2A has been shown to be up-regulated in at least seven types of human cancer [32], our work reports for the first time a rise of PI4KA transcript levels in a human carcinoma. The reason of this up-regulation in HCC remains to be determined. However, studies suggest that PI4KA may regulate signaling pathways involved in survival and proliferation [19].

### Prognostic significance of PI4KA transcript upregulation

**Figure 4:**
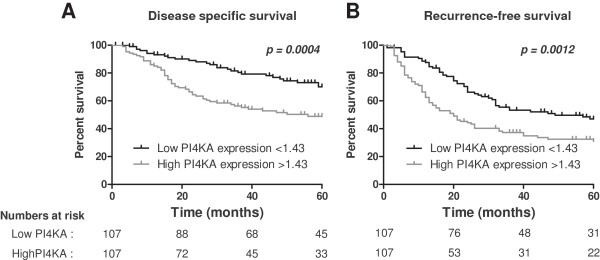
**Mantel-Cox survival curves.** The Kaplan Meier method and Log-Rank test were applied to data for 214 patients with HCC classified into two groups according to whether the PI4KA expression value was above or below the median (low- or high-amount of PI4KA mRNA). **A** and **B** panels present disease-specific survival (tumor-related death) and tumor recurrence-free survival, respectively. *P* values are indicated. Numbers at risk are indicated below each panel.

The annotation of our HCC library includes clinical outcomes, and in particular survival and recurrence data, so we cross-checked these data with PI4KA mRNA expression levels. The 214 HCC samples were subdivided into two groups with respectively low or high amounts of PI4KA mRNA. These groups were stratified by the median value. Patients with highest rate of PI4KA mRNA had shorter disease-specific survival: the first quartile of time to tumor-related death was 16 months for HCC with high PI4KA mRNA level, and 48 months for HCC with low PI4KA mRNA level (*p* = 0.0004, Figure 4A). Similar results were obtained for the risk of tumor recurrence: medians time to tumor recurrence were 20 months for the group with the highest amounts of PI4KA mRNA and 49 months for the other group of patients (p = 0.0012, Figure 4B). Multivariate analysis indicated that high amount of PI4KA mRNA was associated with disease specific survival (HR: 2.36 (1.27; 4.36), *p* = 0.006415), independently of classical clinical, biological and pathological features such as size, number of tumors, microvascular invasion, and tumor portal thrombosis (Table 1). This type of association between PI4KA up-regulation and poor prognosis has been suggested by comparisons of cancer cell lines derived from hamster pancreas: PI4KA was more strongly expressed in the most aggressive cell line [33]. PI4KA could therefore serve as a prognostic marker, in addition to those already identified, and may help improve the accuracy of existing prediction models [20]. Further investigations are required to elucidate the role of this phospholipid kinase in HCC and to assess whether it is a potential targeted for therapy.

**Table 1:** Univariate and multivariate analysis of clinical, pathological and molecular variables for disease-specific survival in 214 HCC

	**Univariate analysis**			**Multivariate analysis**		
**Variables**	**HR**	**95% CI**	***P***	**HR**	**95% CI**	***P***
Cirrhosis	1.45	0.90-2.31	0.119	-	-	-
Multiple tumors	1.952	1.09-3.49	**0.024**	3.53	1.83-6.81	**0.000167**
HCC > 5 cm	1.705	1.04-2.80	**0.035**	1.88	1.01-3.47	**0.044943**
AFP > 20 ng/ml	2.04	1.25-3.32	**0.0043**	1.58	0.90-2.76	0.107818
Microvascular invasion	2.98	1.86-4.78	**5.83e-06**	1.98	1.09-3.60	**0.024328**
Tumor portal thrombosis	3.59	2.14-6.01	**1.21e-06**	1.92	1.01-3.66	**0.045599**
Differentiation: Edmonson III/IV	1.60	1.01-2.52	**0.0436**	0.88	0.50-1.54	0.653579
PI4KA high level	2.22	1.39-3.54	**0.000823**	2.36	1.27-4.36	**0.006415**

Bold values represent *P* values considered to be statistically significant (<0.05).

HR: hazard ratio; CI: confidence interval.

## Conclusions

Our study clearly shows that PI4KA transcripts are more abundant in HCC than normal liver tissue, and that this upregulation is correlated to their differentiation/proliferation status and is associated with poor survival. Further work is needed to determine the involvement, if any, of PI4KA in HCC pathogenesis.

## Abbreviations

HCC: Hepatocellular carcinoma; HBV: Hepatitis B virus; HCV: Hepatitis C virus; HCA: Hepatocellular adenoma; HNF1A: Hepatocyte nuclear factor 1 homeobox A; CTNNB1: Catenin (cadherin-associated protein) beta 1; AFP: Alphafetoprotein; TP53: Tumor protein p53; PI4KA: Phosphatidylinositol 4-kinase type IIIα; PtdIns: Phosphatidylinositol; PLC: Phospholipase C; RT: Reverse transcriptase; PCR: Polymerase chain reaction; ALB: Albumin; ALDOB: Aldolase b; PCNA: Proliferating cell nuclear antigen; MKI67: Antigen identified by monoclonal antibody Ki-67.

## Competing interests

The authors declare that they have no competing interests.

## Authors’ contributions

Conception and design: AI JCN HDPS AC JZR MS JLS. Acquisition of data: AI, JCN, HDPS, JLS. Analysis and interpretation of data: AI JCN HDPS AC JZR MS JLS. Statistical analysis: JCN, JLS. Critical revision of the manuscript for important intellectual content: AI JCN HDPS AC JZR MS JLS. Technical and material support: AI, JCN, HDPS, JLS. Study supervision: AC JZR MS JLS. All authors read and approved the final manuscript.

## Pre-publication history

The pre-publication history for this paper can be accessed here:

http://www.biomedcentral.com/1471-2407/14/7/prepub

## Supplementary Material

Additional file 1 Clinical and molecular features of hepatocellular adenomas. Click here for file

Additional file 2 Characteristics of 217 patients with hepatocellular carcinoma. Click here for file

Additional file 3 Primer sequences. Click here for file

Additional file 4 **Correlation between PI4KA mRNA amount and markers of HCC differentiation status.** Scatter plots show the PI4KA mRNA levels in HCC samples as assayed by RT-real time PCR. Values represent the gene expression of each sample relative to the mean value for two control samples from normal hepatic tissues. The 18S RNA levels were used for normalization. Means with standard deviation are shown for each sample category. *P* values from a Mann-Whitney U-test are indicated. Expression according to TP53 mutation status (NM: not mutated, n = 175; M: mutated, n = 40) (A) or to serum AFP level (AFP < 20 ng/ml, n = 109; AFP > 24 ng/ml, n = 85) (B). Click here for file
