# Supplementary material for: Overexpression of phosphatidylinositol 4-kinase type IIIα is associated with undifferentiated status and poor prognosis of human hepatocellular carcinoma
Source: BMC Cancer. 2014 Jan 6;14:7. doi: 10.1186/1471-2407-14-7 (PMC3898250; doi:10.1186/1471-2407-14-7)
Supplement: Additional file 1 — Clinical and molecular features of hepatocellular adenomas. [file 1471-2407-14-7-S1.doc]

| Parameters | Hepatocellular adenomas  (n = 101) |
| --- | --- |
| Mean age ± SD | 36 ± 10 |
| Female | 82% (83) |
| *HNF1A* mutations | 27% (27) |
| Inflammatory HCA | 44% (44) |
| *CTNNB1* mutations | 10% (10) |
| Inflammatory + CTNNB1 mutations | 13% (13) |
| Non classified HCA | 6% (7) |
| ( ): number of cases |  |
