# Supplementary material for: Overexpression of phosphatidylinositol 4-kinase type IIIα is associated with undifferentiated status and poor prognosis of human hepatocellular carcinoma
Source: BMC Cancer. 2014 Jan 6;14:7. doi: 10.1186/1471-2407-14-7 (PMC3898250; doi:10.1186/1471-2407-14-7)
Supplement: Additional file 2 — Characteristics of 217 patients with hepatocellular carcinoma. [file 1471-2407-14-7-S2.doc]

| Variable | | Total  (n= 217) | |
| --- | --- | --- | --- |
| Age | >60 years | 59% | (128) |
| Gender | Male | 79% | (173) |
| Etiology | HCV | 20% | (43) |
|  | HBV | 21% | (46) |
|  | Alcohol | 36% | (79) |
|  | Hemochromatosis | 9% | (19) |
| Tumor size | < 5 cm | 37% | (81) |
| Tumor number | Single | 87% | (189) |
| Vascular invasion | Microvascular | 41% | (88) |
|  | Macrovascular | 12% | (26) |
| Differentiation | Edmonson I-II | 57% | (118) |
|  | Edmonson III-IV | 43% | (88) |
| Metavir score  (non-tumorous liver) | F0-F1 | 40% | (87) |
|  | F2-F3 | 28% | (60) |
|  | F4 | 31% | (68) |
| Preoperative AFP | > 20 ng/ml | 42% | (92) |
| Child Pugh | A | 96% | (203) |
|  | B | 4% | (9) |
| G1-G6  Classification* | G1 | 9% | (18) |
|  | G2 | 7% | (14) |
|  | G3 | 13% | (26) |
|  | G4 | 36% | (76) |
|  | G5 | 22% | (45) |
|  | G6 | 13% | (28) |
| *CTNNB1* | Mutated | 31% | (68) |
| *TP53* | Mutated | 18% | (40) |
| Events | Median follow up (months) † | 45 (18-60) | |
|  | Tumor death < 5 years | 36% | (78) |
|  | Overall recurrence <5 years | 57% | (122) |
| ( ): number of cases | | | |
| * G1-G6 molecular classification [7] was available for 207 HCC | | | |
| † median (25th and 75th percentile). Follow-up data for survival analysis were available 214 of the 207 HCC. | | | |
