# Supplementary material for: Overexpression of phosphatidylinositol 4-kinase type IIIα is associated with undifferentiated status and poor prognosis of human hepatocellular carcinoma
Source: BMC Cancer. 2014 Jan 6;14:7. doi: 10.1186/1471-2407-14-7 (PMC3898250; doi:10.1186/1471-2407-14-7)
Supplement: Additional file 3 — Primer sequences. [file 1471-2407-14-7-S3.doc]

| Genbank accession nr | Gene | Forward primer (5’-3’) | Reverse primer (5’-3’) |
| --- | --- | --- | --- |
| NM_000477.5 | Albumin | |  | TGCTTGAATGTGCTGATGACAGG | | --- | --- | | AAGGCAAGTCAGCAGGCATCTCATC |
| NM_000035.3 | Aldolase B | GCATCTGTCAGCAGAATGGA | TAGACAGCAGCCAGGACCTT |
| NM_004168.2 | SDHA | TGGGAACAAGAGGGCATCTG | CCACCACTGCATCAAATTCATG |
