# Supplementary figures and images for: Overexpression of phosphatidylinositol 4-kinase type IIIα is associated with undifferentiated status and poor prognosis of human hepatocellular carcinoma
Source: BMC Cancer. 2014 Jan 6;14:7. doi: 10.1186/1471-2407-14-7 (PMC3898250; doi:10.1186/1471-2407-14-7)

**A** TP53 mutation status

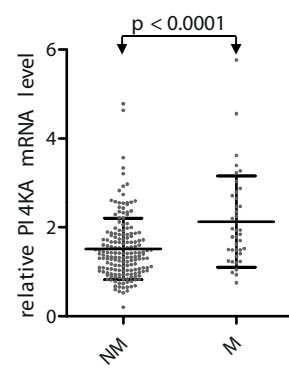

**B** Serum AFP

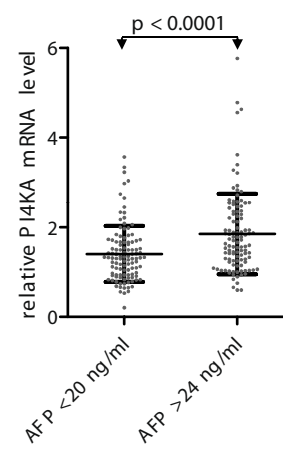

Supplement: Additional file 4 — Correlation between PI4KA mRNA amount and markers of HCC differentiation status. Scatter plots show the PI4KA mRNA levels in HCC samples as assayed by RT-real time PCR. Values represent the gene expression of each sample relative to the mean value for two control samples from normal hepatic tissues. The 18S RNA levels were used for normalization. Means with standard deviation are shown for each sample category. P values from a Mann-Whitney U-test are indicated. Expression according to TP53 mutation status (NM: not mutated, n = 175; M: mutated, n = 40) (A) or to serum AFP level (AFP < 20 ng/ml, n = 109; AFP > 24 ng/ml, n = 85) (B). [file 1471-2407-14-7-S4.pdf]
